# The Combined Impact of Gender and Age on Post-traumatic Stress Symptoms, Depression, and Insomnia During COVID-19 Outbreak in China

**DOI:** 10.3389/fpubh.2020.620023

**Published:** 2021-01-21

**Authors:** Chengbin Liu, Danxia Liu, Ning Huang, Mingqi Fu, Jam Farooq Ahmed, Yanjun Zhang, Xiaohua Wang, Yiqing Wang, Muhammad Shahid, Jing Guo

**Affiliations:** ^1^School of Sociology, Huazhong University of Science and Technology, Wuhan, China; ^2^Center for Social Security Studies, Wuhan University, Wuhan, China; ^3^Department of Anthropology, University of Washington, Seattle, WA, United States; ^4^Department of Anthropology, Quaid-i-Azam University, Islamabad, Pakistan; ^5^School of Social Science, The Chinese University of Hong Kong, Hong Kong, China; ^6^School of Social Development and Public Policy, Beijing Normal University, Beijing, China; ^7^World Health Organization, Balochistan, Pakistan; ^8^Department of Health Policy and Management, School of Public Health, Peking University, Beijing, China

**Keywords:** PTSD, depression, insomnia, age, gender, China

## Abstract

The mental health problems might have been increased owing to the COVID-19 pandemic with the commencement of the year 2020, therefore, an epidemiological survey appraising the burden of mental health issues among the general population is imperative. This cross-sectional study attempts to reveal the underlying mental health conditions, such as Post-Traumatic Stress Symptoms (PTSS), depression, and insomnia, relating to the pandemic situation, and to further examine the combined effects of gender and age on the COVID-19 related mental health consequences. An online survey was conducted among 2,992 adults in China from February 1st 2020 to February 10th 2020. The study uses binary logistic regression to analyze the potential factors associated with PTSD, depression, and insomnia. The results indicate that the prevalence of PTSS, depression, and insomnia are 19.5, 26.9, and 19.6% respectively during the COVID-19. Men and women show different rates of PTSS and depression, whereas no insomnia is found in both males and females. The females above 50 years of age have a lower level of depressive symptoms (OR = 0.448, 95%CI: 0.220–0.911, Cohen's d = −0.443) as compared with females aged 18–25; while the highest effect sizes for PTSS (OR = 2.846, 95%CI: 1.725–4.695, Cohen's d = 0.537) and the depression (OR = 2.024, 95%CI: 1.317–3.111, Cohen's d = 0.314) are seen in males aged 26 to 30. Besides gender, education, living conditions, direct exposure to COVID-19, the post mental and the physical health condition is related to PTSS, depression, and insomnia. Our study suggests that high-risk groups, especially those having two or more related factors and young men, should be the focus of mental health intervention.

## Introduction

Detected by the end of 2019, Corona Virus Disease, known as COVID-19, has become a global pandemic now after affecting millions of people worldwide. The outbreak and the spread of COVID-19 caused multiple challenges relating not only to political management, economic growth, and healthcare delivery on the macro-level but also to the psychological well-being of individuals (1, 2).

Recently, anxiety, depression, insomnia, denial, anger, and fear among medical workers in Wuhan can be observed, associated with excessive work burden and intensive dangers of contagious infection (3, 4). However, as a new form of a stressor for mental health (5), the COVID-19 pandemic affects populations beyond healthcare workers. Unlike natural disasters that have specific regional impacts in a given time (6), the impact of this global crisis is profound and lasting. The social risks in the COVID-19 pandemic are not as recognizable as those in wars or international mass conflicts (7). One meta-analysis study suggests over one-in-five people experienced post-traumatic stress symptoms (PTSS) and psychological stress (8). Another systematic review indicates that the general population in many countries reported a relatively high prevalence of depression, posttraumatic stress disorder, psychological distress during the COVID-19 pandemic (9). Thus, it is rational to assume that this epidemic is sweeping across the population, and an epidemiological survey of the general population is essential for evaluating the actual mental health burden of the COVID-19 crisis.

For mental health studies, gender and age are primarily considered as demographic variables and get less attention as such. Neither gender nor age is the main area to focus on within most mental health studies. As early as Freud, gender differences were recognized in mental health because women were believed to be stunted in both ego and superego development, further resulting in passivity as a gender characteristic (10). This idea was later criticized by Rosenfield and Smith, claiming that there were no differences in the overall rates of psychopathy between genders, but admitted that males and females differ in the type of psychopathology experiences (11). Females develop more internalizing disorders, even though they are less subjected to potentially traumatic events (12). However, male counterparts have higher rates of externalizing problems. The variation in the extent of gender differences on mental health varies between trauma types (13). However, it should be noted that the evidence during the pandemic context is lacking.

Moreover, as noted by gender-roles theory, males and females show differences in the age distribution of mental health issues during their life course (14). Additionally, gender is found to be a significant biomarker of brain development and behavioral development throughout the lifespan so that it has further interactions with the mental health of individuals (15). But, how exactly gender and age affect mental health under traumatic circumstances is not clear. Taking post-traumatic stress disorder (PTSD) as an internalizing disorder, Kessler's study demonstrates that there is no age difference for men across age groups, despite a tendency for PTSD symptoms to decline as women get older (16). On the contrary, another study suggests that females of 25–35 and males between 45 and 55 years might suffer the highest level of PTSS (17), possibly due to changes in sympathetic or noradrenergic systems (18). Besides, the study of Norris shows that women aged between 55 and 64 years old are most possibly to suffer PTSD symptoms (19). Some other studies claim that it is more likely for individuals aged between 18 and 24 years to get PTSD symptoms (20). The inconsistency in these studies could be attributed to methodological or cultural differences, and this situation, therefore, suggests urgency for more evidence highlighting how epidemics in a social setting may affect the mental health risk evaluation as an important factor.

Influenced by ancient Confucian traditions and current market expansion, “males are considered the main breadwinners while females are the primary caretakers” in China (21). Chinese men as the primary supporter for the family may undergo more stress facing higher psychological symptoms owing to the economic ebb and the higher COVID-19 related mortality rate (22). According to the life course theory, there is an inverted U-shape between mental health symptoms and age. The highest symptoms may be in young adulthood and decrease after midlife (23). The stress about the job, parenting young children, and marriage is very common in early adulthood but it diminishes with time, however, health problems are a major cause of stress in late adulthood (24). Therefore, Chinese young adults with jobs and married status may have the highest psychological symptoms during the pandemic. Combining the gender role theory and life course theory, we expect that young males may have higher financial stress regarding supporting their family as compared with young females.

During the COVID-19 crisis, especially with social distancing measures and policies to slow the spreading speed, PTSD, depression, and insomnia are the three most prevalent psychiatric disorders affecting the individuals' mental health (25, 26). In addition to gender and age relationship with PTSS, depression, and insomnia, previous studies on pandemics have found other potential factors including the personal characteristics, the traumatic exposure, the individuals' physical health and the psychological states, and so on (9). However, the significance of those factors varies in different psychiatric studies. One study estimates the prevalence of PTSD which is 7% in COVID-19 hard-hit areas in China, while gender, exposure history, and sleep quality also matter (27). Other studies suggest a 16.5–17.7% prevalence of depression, while the predictions from gender, age, educational levels, and professions are significant (28, 29). However, none of these studies focus on the related factors of PTSS, depression, insomnia simultaneously. To identify the shared factors and the specific factors of PTSS, depression, and insomnia, this study, therefore, attempts to discuss three typical symptoms in unison to allocate the limited resources more effectively.

For the reasons discussed above, the objectives of this study are (1) to estimate the prevalence of PTSD symptoms, depression, and insomnia among the general population during the COVID-19 outbreak; (2) to examine the combined effect of gender and age on PTSD symptoms, depression, and insomnia respectively; (3) to figure out the shared factors and the specific factors which are associated with PTSS, depression, and insomnia. Based on the reviewed literature, three hypotheses are proposed for the current study. First, we expected that males may have higher symptoms of PTSD, depression, and insomnia than females in china during the COVID-19 outbreak. Second, we assumed that young adults may experience higher PTSS, depression, and insomnia symptoms. Third, we proposed that age may have a significant interaction effect with the gender on PTSS, depression, and insomnia. Lastly, we expected that there exist other factors like the living conditions, the direct exposure to COVID-19, the post mental, and the physical health condition associated with PTSS, depression, and insomnia.

## Methods

### Data Source, Procedure, and Participants

This survey was conducted online from February 1st to February 10th in 2020, and all questionnaires were given out and retrieved through a web-based platform (https://www.wjx.cn/app/survey.aspx). In total, 2,992 participants across 31 Chines provinces participated in the survey. A snowball sampling was used to select participants and Chinese citizens aged ≥ 18 years old were invited. To reach more subjects from groups with high exposure to COVID-19 and low social-economic status, we sent out questionnaires to some specific citizens. After excluding 134 questionnaires of low quality (excluding criteria including finishing in shorter than 10 min or having some logical problem et al.), we finally got 2,858 subjects, including medical workers (*N* = 421, 14.7%), nonprofessional employees (*N* = 259, 9.1%), social service workers (*N* = 230, 8.0%), teachers and faculties (*N* = 648, 22.7%), workers and farmers (*N* = 388, 13.6%), students (*N* = 424, 14.8%), unemployed and others (*N* = 488, 17.1%). All participants gave their consent and joined this research voluntarily after being informed about the nature of the study. This study was approved by the Ethics Committee of the Peking University Medical Center.

### Measures

**Depression** was assessed with the help of a 20-item scale used by the Center for Epidemiological Studies Depression (CESD) to measure depressive symptoms in the general population (30). Previous studies have proved that this scale has high reliability and validity among Chinese (31). Respondents reported the frequency of each symptoms item on a four-point scale: 0 (rarely or none of the time; <1 day), 1 (some of the time; 1–2 days), 2 (much or a moderate amount of the time; 3–4 days), or 3 (most or all of the time; 5–7 days). The total score ranges from 0 to 60, with a higher score indicating a higher level of depressive symptoms. With a cut-off point at 21, respondents were divided into two categories, “depressed” or “no depressive symptoms.” Cronbach's alpha was 0.93 in this study.

**PTSS** was assessed by a 20-item self-report PCL-5 (PTSD Checklist for DSM-5) scale, estimating the degree to which individuals have been disturbed in the past month using PTSD symptoms (32). Respondents answered 20 items on a four-point scale rating from 0 (not at all) to 4 (extremely). Items were summed for a total score ranging from 0 to 80, with higher scores indicating a higher level of PTSS. Each item rated at least 2 (moderate) could be regarded as PTSD symptoms. And 20 items were divided into four DSM-5 PTSD symptoms clusters: intrusions (items 1–5), avoidance (items 6–7), negative alterations in mood and cognition (items 8–14), alterations in reactivity, and arousal (items 15–20). The diagnostic criteria of DSM-5 required at least 1 “*intrusions-symptom*,” 1 “*avoidance-symptom*,” 2 “*negative alterations in the mood and the cognition-symptoms*,” and 2 “*alterations in reactivity and arousal-symptoms*.” The Cronbach's alpha was 0.97 in this study.

**Insomnia** was estimated with The Pittsburgh Sleep Quality Index (PSQI) (33). The PSQI (Chinese Version) was translated and validated by Liu and associates (34). The PSQI is constitutive of 19 self-reported items including various factors about sleep quality consisting of estimation of sleep latency, duration, disturbances, and the severity and frequency of other sleep problems. The total PSQI scale is grouped into seven 0–3 subscales, with the total score ranging from 0 to 21 and higher scores indicating worse sleep quality. With a cut-off point at 7, respondents were divided into two categories, “insomnia” or “no insomnia.” The Cronbach's alpha in this study was 0.86.

**Exposure items** included Wuhan exposure (“1” refers to lived or had Wuhan travel history, “0” refers to none Wuhan travel history), prior exposure (yes, no), media exposure (frequently, sometimes, less, very less), impact on livelihood (none, some, relatively large, very large) and direct exposure to COVID-19 (“1” includes self, family, friend, and neighborhood exposure to COVID-19, “0” refers to none exposure).

Gender in this study was divided into males and females, and age was categorized as 18–25, 26–30, 31–40, 41–50, 51, and over comprehensively considering the basic age distribution and the internal variation between age groups. Also, socioeconomic covariates in this study include ethnicity (Han, else), marriage (have no spouse, have a spouse), education (junior high school and below, high school/technical school, junior college, undergraduate, postgraduate and above), job (medical workers, nonprofessional employees, social service workers, teachers and operators, students, workers and farmers, unemployed and others) and income (poor, not poor). Health-related variables contained prior and post psychological problems (yes, no), chronic diseases (yes, no), and 2-week illness (yes, no). These variables are included in the study according to previous studies (23, 24).

### Statistical Analyses

Descriptive analysis was conducted to describe the characteristics of the sample. In the analyses, PTSS, depression, and insomnia were used as binary variables. χ^2^ or *t*-test was used to examine the binary correlation between independent variables with PTSS, depression, insomnia respectively. Then, three logistic regression models were used to examine the factors linked to PTSS, depression, and insomnia. Finally, another two logistic regression models were designed to examine the combined effect of gender and age on PTSS and depression. All potentially confounding variables including socio-demographic variables (consisted of ethnicity, marriage, education, job, and income), health-related factors (contained prior and post psychological problems, chronic diseases, and 2-week illness), were controlled in the above models. We set the alpha at 0.05 for statistical significance in all the tests. SPSS 22.0 was used to carry out these analyses.

## Results

### Descriptive Analyses

As shown in Table 1, about 95.8% of the total 2,858 participants belong to the Han ethnicity, and the proportion of men and women is nearly equal (46.4% as male and 53.6% as female). The distribution of age groups is presented as following: participants aged 31–40 years constitute the most (about 31.2%), followed by those aged 18–25 years (about 24.2%), aged 26–30 years (about 22.6%), and aged 41–50 years (about 14.0%); participants above 50 years of age contribute to merely 8.1% of the sample. Besides, 60.2% of the participants are married and nearly 60% of them are well-educated (undergraduate or above). When it comes to the traumatic exposure, there are 85.5% of participants considering themselves as being free of the Wuhan exposure and about 92.1% of the samples are out of prior traumatic exposures. However, nearly 83% of the participants are under indirect exposure to COVID-19, occasionally or frequently through media in particular. In general, the health condition of most participants is good, as the proportion for participants having the prior psychological problem, the post psychological problem, the chronic diseases, and the 2-week illness are 14.6, 29, 12, and 7% respectively. More detailed, among all 2,858 participants, 19.5% are found of PTSS, 26.9% of depression, and 19.6% of insomnia. More details could be seen in Table 1.

**Table 1:** Descriptive analysis of sample characteristics.

	**Total**		**Male**		**Female**		***P*-value**
	***N***	**%**	***N***	**%**	***N***	**%**	
**PTSS**							*p* < 0.001
Yes	558	19.5	334	25.2	224	14.6	
No	2,300	80.5	992	74.8	1,308	85.4	
**Depression**							*p* < 0.001
<21	2,088	73.1	897	67.6	1,191	77.7	
≥21	770	26.9	429	32.4	341	22.3	
**Sleep quality**							0.001
≤7	2,297	80.4	1,030	77.7	1,267	82.7	
>7	561	19.6	296	22.3	265	17.3	
**Ethnicity**							0.070
Han	2,738	95.8	1,280	96.5	1,458	95.2	
Else	120	4.2	46	3.5	74	4.8	
**Gender**							
Male	1,326	46.4					
Female	1,532	53.6					
**Age**							0.027
18–25	691	24.2	309	23.3	382	24.9	
26–30	645	22.6	272	20.5	373	24.3	
31–40	891	31.2	425	32.1	466	30.4	
41–50	400	14.0	200	15.1	200	13.1	
≥51	231	8.1	120	9.0	111	7.2	
**Marriage**							0.672
Not have a spouse	1,137	39.8	552	41.6	615	40.1	
Have a spouse	1,721	60.2	804	60.6	917	59.9	
**Education**							*p* < 0.001
Junior high school and below	268	9.4	127	9.6	141	9.2	
High school/Technical school	387	13.5	231	17.4	156	10.2	
Junior College	488	17.1	247	18.6	241	15.7	
Undergraduate	1,257	44.0	559	42.2	698	45.6	
Postgraduate and above	458	16.0	162	12.2	296	19.3	
**Job**							*p* < 0.001
Medical workers	421	14.7	88	6.6	333	21.7	
Nonprofessional employees	259	9.1	174	13.1	85	5.5	
Social service workers	230	8.0	129	9.7	101	6.6	
Teachers and operators	648	22.7	304	22.9	344	22.5	
Students	424	14.8	169	12.7	255	16.6	
Workers and farmers	388	13.6	244	18.4	144	9.4	
Unemployed and others	488	17.1	218	16.4	270	17.6	
**Income**							*p* < 0.001
Poor	327	11.4	200	15.1	127	8.3	
Not poor	2,531	88.6	1,126	84.9	1,405	91.7	
**Wuhan exposure**							0.002
Yes	413	14.5	163	12.3	250	16.3	
No	2,445	85.5	1,163	87.7	1,282	83.7	
**Impact on livelihood**							0.055
None	825	28.9	358	27.0	467	30.5	
Some	975	34.1	454	34.2	521	34.0	
Relatively large	611	21.4	284	21.4	327	21.3	
Very large	447	15.6	230	17.3	217	14.2	
**Prior exposure**							0.229
Yes	227	7.9	114	8.6	113	7.4	
No	2,631	92.1	1,212	91.4	1,419	92.6	
**Media exposure**							0.125
Frequently	1,608	56.3	759	57.2	849	55.4	
Sometimes	762	26.7	328	24.7	434	28.3	
Less	259	9.1	131	9.9	128	8.4	
Very less	229	8.0	108	8.1	121	7.9	
**Prior psychological problems**							0.292
Yes	418	14.6	184	13.9	234	15.3	
No	2,440	85.4	1,142	86.1	1,298	84.7	
**Post psychological problems**							0.003
Yes	828	29.0	348	26.2	480	31.3	
No	2,030	71.0	978	73.8	1,052	68.7	
**Chronic disease**							0.701
Yes	342	12.0	162	12.2	180	11.7	
No	2,516	88.0	1,164	87.8	1,352	88.3	
**Two-week illness**							0.359
Yes	201	7.0	87	6.6	114	7.4	
No	2,657	93.0	1,239	93.4	1,418	92.6	
	Mean	SD	Mean	SD	Mean	SD	
Direct exposure	0.6	1.2	0.5	1.1	0.6	1.3	0.035

To identify possible factors associated with mental disorders, this study further conducts binary analysis, where results are presented in Table 2. Findings indicated that PTSS, depression, and insomnia share some factors in common, including gender, age, education, profession, income, psychological health conditions, and the 2-week illness, as well as impacts of COVID-19 on livelihood and traumatic exposure experiences. However, there are some characteristics with partial significance. For example, the different marital status affects PTSS and insomnia only, and suffering from chronic diseases is related only to higher depressive symptoms. Also, people who live in Wuhan or even have been to Wuhan within 2 weeks before the outbreak of COVID-19 would reflect the higher level of insomnia, but prior exposure experiences are insignificantly related. More details are presented in Table 2.

**Table 2:** Binary correlations of risk factors with PTSS, depression, sleep quality.

	**PTSS** ***N*** **(%)**			**Depression** ***N*** **(%)**			**Insomnia** ***N*** **(%)**		
	**Yes**	**No**	***P*-value**	**Yes**	**No**	***P*-value**	**>7**	**≤7**	***P*-value**
**Ethnicity**									
Han	538 (19.6)	2,200 (80.4)	0.420	739 (27.0)	1,999 (73.0)	0.780	538 (19.6)	2,200 (80.4)	0.896
Else	20 (16.7)	100 (83.3)		31 (25.8)	89 (74.2)		23 (19.2)	97 (80.8)	
**Gender**									
Male	334 (25.2)	992 (74.8)	*p* < 0.001	429 (32.4)	897 (67.6)	*p* < 0.001	296 (22.3)	1,030 (77.7)	0.001
Female	224 (14.6)	1,308 (85.4)		341 (22.3)	1,191 (77.7)		265 (17.3)	1,267 (82.7)	
**Age**									
18–25	124 (17.9)	567 (82.1)	0.006	190 (27.5)	501 (72.5)	*p* < 0.001	113 (16.4)	578 (83.6)	0.003
26–30	143 (22.2)	502 (77.8)		184 (28.5)	461 (71.5)		112 (17.4)	533 (82.6)	
31–40	193 (21.7)	698 (78.3)		266 (29.9)	625 (70.1)		207 (23.2)	684 (76.8)	
41–50	68 (17.0)	332 (83.0)		94 (23.5)	306 (76.5)		76 (19.0)	324 (81.0)	
≥51	30 (13.0)	201 (87.0)		36 (15.6)	195 (84.4)		53 (22.9)	178 (77.1)	
**Marriage**									
Not have a spouse	194 (17.1)	943 (82.9)	0.007	292 (25.7)	845 (74.3)	0.217	188 (16.5)	949 (83.5)	0.001
Have a spouse	364 (21.2)	1,357 (78.8)		478 (27.8)	1,243 (72.2)		373 (21.7)	1,348 (78.3)	
**Education**									
Junior high school and below	45 (16.8)	223 (83.2)	*p* < 0.001	62 (23.1)	206 (76.9)	*p* < 0.001	52 (19.4)	216 (80.6)	*p* < 0.001
High school/Technical school	111 (28.7)	276 (71.3)		139 (35.9)	248 (64.1)		99 (25.6)	288 (74.4)	
Junior College	108 (22.1)	380 (77.9)		135 (27.7)	353 (72.3)		110 (22.5)	378 (77.5)	
Undergraduate	240 (19.1)	1,017 (80.9)		337 (26.8)	920 (73.2)		235 (18.7)	1,022 (81.3)	
Postgraduate and above	54 (11.8)	404 (88.2)		97 (21.2)	361 (78.8)		65 (14.2)	393 (85.8)	
**Job**									
Medical workers	66 (15.7)	355 (84.3)	*p* < 0.001	103 (24.5)	318 (75.5)	0.002	102 (24.2)	319 (75.8)	0.005
Nonprofessional employees	80 (30.9)	179 (69.1)		96 (37.1)	163 (62.9)		52 (20.1)	207 (79.9)	
Social service workers	44 (19.1)	186 (80.9)		57 (24.8)	173 (75.2)		48 (20.9)	182 (79.1)	
Teachers and operators	131 (20.2)	517 (79.8)		164 (25.3)	484 (74.7)		127 (19.6)	521 (80.4)	
Students	64 (15.1)	360 (84.9)		105 (24.8)	319 (75.2)		60 (14.2)	364 (85.8)	
Workers and farmers	91 (23.5)	297 (76.5)		119 (30.7)	269 (69.3)		89 (22.9)	299 (77.1)	
Unemployed and others	82 (16.8)	406 (83.2)		126 (25.8)	362 (74.2)		83 (17.0)	405 (83.0)	
**Income**									
Poor	88 (26.9)	239 (73.1)	*p* < 0.001	109 (33.3)	218 (66.7)	0.006	84 (25.7)	243 (74.3)	0.003
Not poor	470 (18.6)	2,061 (81.4)		661 (26.1)	1,870 (73.9)		477 (18.8)	2,054 (81.2)	
**Wuhan exposure**									
Yes	69 (16.7)	344 (83.3)	0.118	116 (28.1)	297 (71.9)	0.571	96 (23.2)	317 (76.8)	0.046
No	489 (20.0)	1,956 (80.0)		654 (26.7)	1,791 (73.3)		465 (19.0)	1,980 (81.0)	
**Impact on livelihood**									
None	90 (10.9)	735 (89.1)	*p* < 0.001	148 (17.9)	677 (82.1)	*p* < 0.001	131 (15.9)	694 (84.1)	*p* < 0.001
Some	160 (16.4)	815 (83.6)		231 (23.7)	744 (76.3)		170 (17.4)	805 (82.6)	
Relatively large	177 (29.0)	434 (71.0)		224 (36.7)	387 (63.3)		143 (23.4)	468 (76.6)	
Very large	131 (29.3)	316 (70.7)		167 (37.4)	280 (62.6)		117 (26.2)	330 (73.8)	
**Prior exposure**									
Yes	59 (26.0)	168 (74.0)	0.010	78 (34.3)	149 (65.6)	0.009	50 (22.0)	177 (78.0)	0.343
No	499 (19.0)	2,132 (81.0)		692 (26.3)	1,939 (73.7)		511 (19.4)	2,120 (80.6)	
**Media exposure**									
Frequently	346 (21.5)	1,262 (78.5)	0.005	451 (28.0)	1,157 (72.0)	0.035	346 (21.5)	1,262 (78.5)	0.029
Sometimes	119 (15.6)	643 (84.4)		184 (24.1)	578 (75.9)		126 (16.5)	636 (83.5)	
Less	54 (20.8)	205 (79.2)		82 (31.7)	177 (68.3)		49 (18.9)	210 (81.1)	
Very less	39 (17.0)	190 (83.0)		53 (23.1)	176 (76.9)		40 (17.5)	189 (82.5)	
**Prior psychological problems**									
Yes	126 (30.1)	292 (69.9)	*p* < 0.001	204 (48.8)	214 (51.2)	*p* < 0.001	137 (32.8)	281 (67.2)	*p* < 0.001
No	432 (17.7)	2,008 (82.3)		566 (23.2)	1,874 (76.8)		424 (17.4)	2,016 (82.6)	
**Post psychological problems**									
Yes	247 (29.8)	581 (70.2)	*p* < 0.001	355 (42.9)	473 (57.1)	*p* < 0.001	244 (29.5)	584 (70.5)	*p* < 0.001
No	311 (15.3)	1,719 (84.7)		415 (20.4)	1,615 (79.6)		317 (15.6)	1,713 (84.4)	
**Chronic disease**									
Yes	61 (17.8)	281 (82.2)	0.401	113 (33.0)	229 (67.0)	0.007	100 (29.2)	242 (70.8)	*p* < 0.001
No	497 (19.8)	2,019 (80.2)		657 (26.1)	1,859 (73.9)		461 (18.3)	2,055 (81.7)	
**Two-week illness**									
Yes	63 (31.3)	138 (68.7)	*p* < 0.001	96 (47.8)	105 (52.2)	*p* < 0.001	75 (37.3)	126 (62.7)	*p* < 0.001
No	495 (18.6)	2,162 (81.4)		674 (25.4)	1,983 (74.6)		486 (18.3)	2,171 (81.7)	
	Mean (SD)		*P* value	Mean (SD)		*P* value	Mean (SD)		*P*-value
Direct exposure	0.8 (1.6)	0.5 (1.1)	*p* < 0.001	0.9 (1.6)	0.5 (1.0)	*p* < 0.001	1.0 (1.7)	0.5 (1.0)	*p* < 0.001

### Logistic Regression Analyses

As shown in Table 3, the prevalence of PTSS is generally higher among males than females (OR = 1.824, 95%CI: 1.477–2.251, Cohen's d = 0.331). In comparison with single and above 50-year-old participants, those aged between 26 and 30 years and married possibly suffer from higher PTSS (OR = 1.796, 95%CI: 1.103, 2.925, Cohen's d = 0.323). Essential service jobs, direct exposure to COVID-19, negative impact on livelihood, post psychological problems, 2-week illness are significantly associated with a higher level of PTSS. Counter-intuitively, participants with higher education, the Wuhan contact, and sometimes media exposure are less likely to be diagnosed with PTSD.

**Table 3:** Logistic regression analysis for risk factors of PTSS, depression and insomnia.

**Variables**	**Model 1-PTSS**		**Model 2-Depression**		**Model 3-Insomnia**	
	**OR (95% CI)**	**Cohen's d**	**OR (95% CI)**	**Cohen's d**	**OR (95% CI)**	**Cohen's d**
**Wuhan exposure (No)**						
Yes	0.694^*^ **(0.501, 0.961)**	−0.201	0.883 (0.668, 1.168)	−0.069	0.995 (0.739, 1.340)	−0.003
**Impact on livelihood (None)**						
Some	1.499^**^ **(1.123, 1.999)**	0.223	1.393^**^ **(1.089, 1.781)**	0.183	1.146 (0.882, 1.490)	0.075
Relatively large	3.054^***^ **(2.275, 4.101)**	0.616	2.482^***^ **(1.914, 3.218)**	0.051	1.579^**^ **(1.193, 2.089)**	0.252
Very large	2.590^***^ **(1.879, 3.571)**	0.525	2.255^***^ **(1.693, 3.003)**	0.448	1.632^**^ **(1.202, 2.216)**	0.270
**Prior exposure (No)**						
Yes	1.204 (0.851, 1.705)	0.102	1.068 (0.772, 1.477)	0.036	0.789 (0.548, 1.134)	−0.131
Direct exposure	1.186^**^ **(1.069, 1.315)**	0.094	1.187^**^ **(1.077, 1.308)**	0.095	1.257^***^ (1.138, 1.389)	0.126
**Media exposure (Frequently)**						
Sometimes	0.768^*^ **(0.601, 0.981)**	−0.146	0.941 (0.758, 1.168)	−0.034	0.793 (0.625, 1.007)	−0.128
Less	0.936 (0.656, 1.333)	−0.036	1.298 (0.947, 1.778)	0.144	0.863 (0.605, 1.231)	−0.081
Very less	0.813 (0.546, 1.210)	−0.114	0.915 (0.638, 1.312)	−0.049	0.807 (0.547, 1.191)	−0.118
**Ethnicity (Han)**						
Else	0.919 (0.546, 1.545)	−0.047	1.005 (0.918, 1.101)	0.003	0.969 (0.590, 1.591)	−0.017
**Gender (Female)**						
Male	1.824^***^ **(1.477, 2.251)**	0.331	1.698^***^ **(1.405, 2.052)**	0.292	1.390^**^ **(1.131, 1.707)**	0.182
**Age (≥51)**						
18–25	1.471 (0.846, 2.559)	0.213	2.245^**^ **(1.348, 3.739)**	0.446	0.714 (0.432, 1.179)	−0.186
26–30	1.796^*^ **(1.103, 2.925)**	0.323	2.369^***^ **(1.500, 3.739)**	0.476	0.718 (0.465, 1.106)	−0.183
31–40	1.419 (0.894, 2.253)	0.193	2.166^***^ **(1.407, 3.333)**	0.426	0.965 (0.652, 1.430)	−0.020
41–50	1.124 (0.679, 1.860)	0.064	1.631^**^ **(1.024, 2.597)**	0.270	0.761 (0.493, 1.174)	−0.151
**Marriage (None spouse)**						
Have a spouse	1.368^**^ **(1.022, 1.831)**	0.173	1.212 (0.931, 1.577)	0.106	1.050 (0.789, 1.398)	0.027
**Education (Postgraduate and above)**						
Junior high school and below	1.540 (0.933, 2.540)	0.238	1.251 (0.807, 1.939)	0.123	1.471 (0.912, 2.371)	0.213
High school/Technical school	2.373^**^ **(1.573, 3.581)**	0.476	1.818^**^ **(1.268, 2.607)**	0.330	2.028^***^ **(1.364, 3.016)**	0.390
Junior College	1.940^**^ **(1.305, 2.885)**	0.365	1.379 (0.979, 1.943)	0.177	1.901^**^ **(1.304, 2.773)**	0.354
Undergraduate	1.679^**^ **(1.193, 2.363)**	0.286	1.309 (0.985, 1.739)	0.148	1.351 (0.978, 1.867)	0.166
**Job (Medical workers)**						
Nonprofessional employees	1.721^*^ **(1.129, 2.621)**	0.299	1.421 (0.967, 2.089)	0.194	0.643^*^ **(0.421, 0.982)**	−0.243
Social service workers	1.488 (0.938, 2.358)	0.219	1.175 (0.777, 1.777)	0.089	0.978 (0.641, 1.492)	−0.012
Teachers and operators	1.335 (0.927, 1.921)	0.159	1.032 (0.747, 1.426)	0.017	0.757 (0.544, 1.054)	−0.153
Students	1.231 (0.752, 2.017)	0.115	1.030 (0.669, 1.587)	0.016	0.647 (0.402, 1.042)	−0.240
Workers and farmers	1.346 (0.890, 2.037)	0.164	1.290 (0.890, 1.871)	0.140	0.804 (0.546, 1.182)	−0.120
Unemployed and others	1.036 (0.699, 1.535)	0.019	1.108 (0.787, 1.559)	0.057	0.629^*^ **(0.438, 0.903)**	−0.256
**Income (Not poor)**						
Poor	1.276 (0.953, 1.709)	0.134	1.098 (0.834, 1.447)	0.052	1.377^*^ **(1.028, 1.846)**	0.176
**Prior psychological problems (No)**						
Yes	1.316 (0.992, 1.745)	0.151	1.930^***^ **(1.498, 2.486)**	0.363	1.572^**^ **(1.199, 2.062)**	0.249
**Post psychological problems (No)**						
Yes	2.026^***^ **(1.609, 2.552)**	0.389	2.168^***^ **(1.762, 2.668)**	0.427	1.658^***^ **(1.321, 2.080)**	0.279
**Chronic disease (No)**						
Yes	0.741 (0.528, 1.039)	−0.165	1.204 (0.904, 1.602)	0.102	1.412^*^ **(1.058, 1.884)**	0.190
**Two-week illness (No)**						
Yes	1.554^*^ **(1.074, 2.248)**	0.243	1.829^***^ **(1.303, 2.566)**	0.333	1.766^**^ **(1.249, 2.497)**	0.314

The values of coefficients and 95% confidence interval in bold represent statistically significant at 0.05 level.

* p <0.05,

** p <0.01,

*** *p <0.001*.

Factors correlated with depression are mostly similar to those for PTSS, however, a few differences ought to be noted. Firstly, significant differences exist between all age groups. Take people aged over 51 as a reference, those aged 18–50 are more likely to be depressed. In detail, the Cohen's d effect size is highest in the 26–30 age group, followed by the 18–25 age group and 31–40 age group, while is lowest in the 41–50 age group. And the Cohen's d values of all these age groups are over 0.2 and below 0.5, indicating a medium association with depression. Secondly, participants with prior psychological problems, high school/technical school education, post psychological problems, and 2-week illness incline to a higher level of depression. And the Cohen's d effect sizes of all these variables are medium (over 0.2 and below 0.5).

When it comes to insomnia, there exists a significant gender variation in the PTSS prevalence (OR = 1.390, 95%CI: 1.131–1.707, Cohen's d = 0.182), but no age differences. Compared with medical workers who are intensively exposed, individuals in essential service jobs and those being unemployed are less possibly to experience PTSS, and both the Cohen's d effect sizes of them were medium. And people suffering from chronic diseases may be more prone to have high insomnia symptoms (OR = 1.412, 95%CI: 1.058–1.884, Cohen's d = 0.190), although Cohen's d effect size is small.

**Figure 1:**
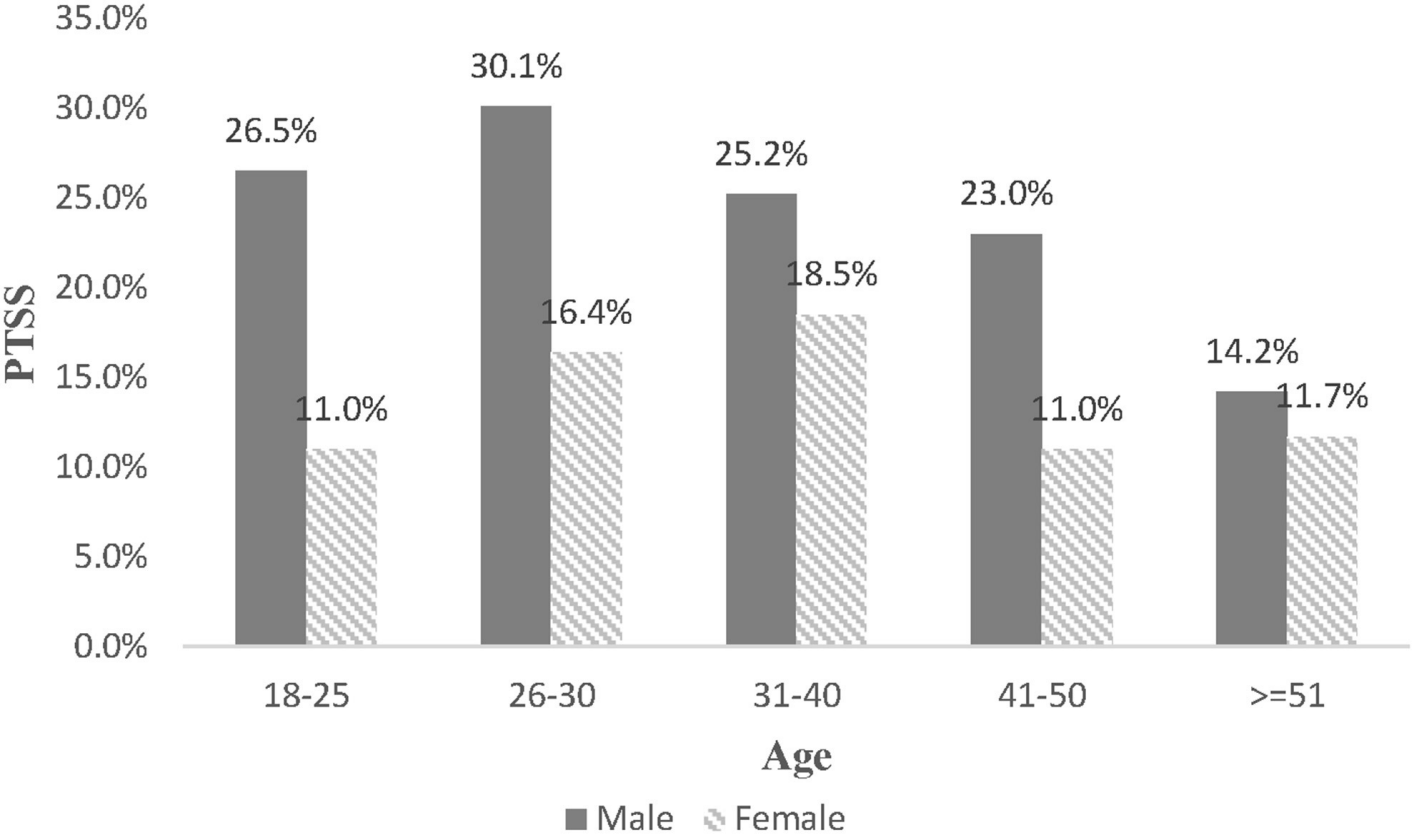
The combine effect of gender and age on PTSS.

**Figure 2:**
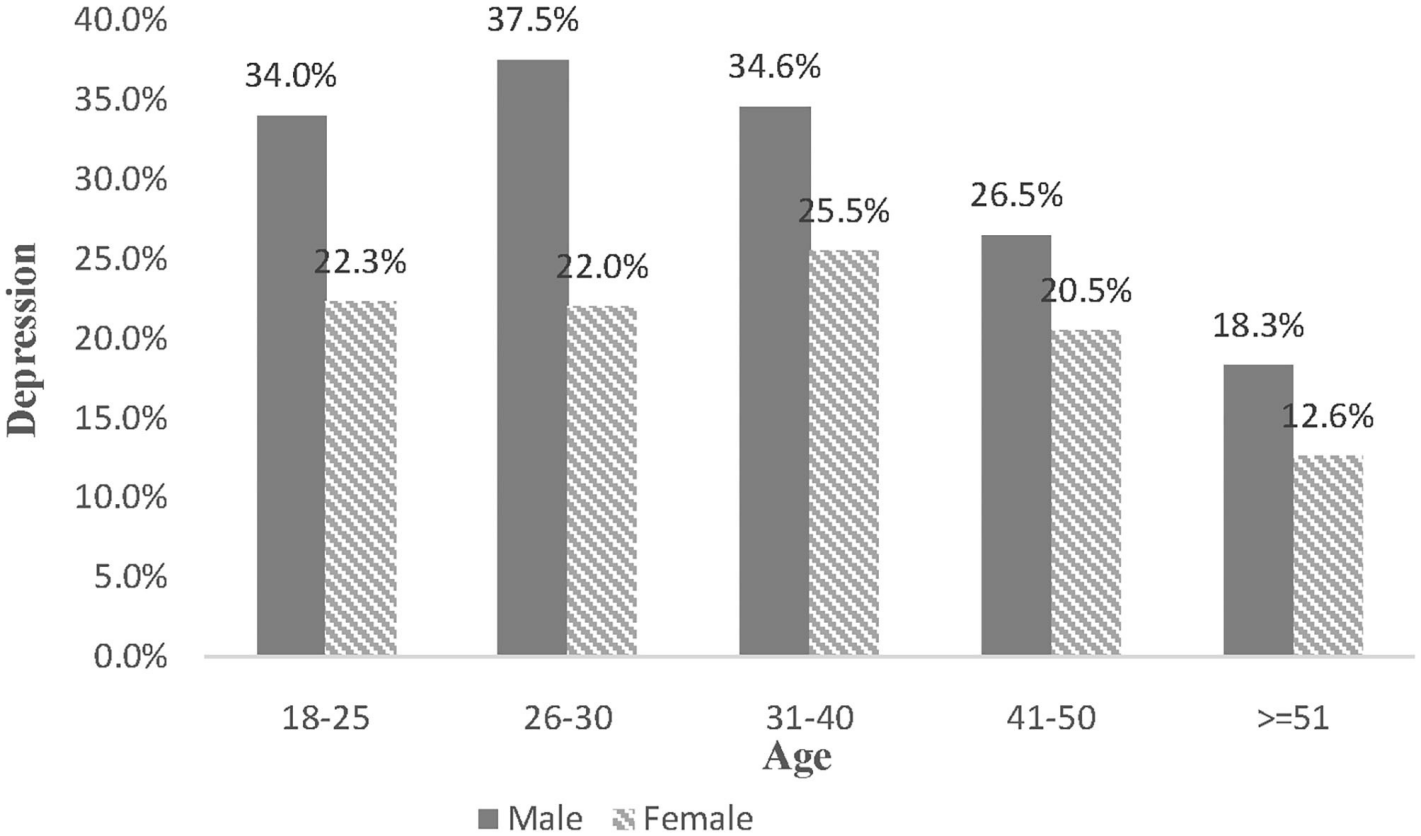
The combine effect of gender and age on depression.

Since age has an insignificant association with insomnia, this study further examines the combined effect of gender and age on PTSS and depression. Although no significant differences are found among other age groups, men aged 18–50 may experience a high degree of PTSS, compared with females aged 18–25 years old. At the same time, the age distribution of depressive prevalence is different (see Figure 2). Despite no differences exist between females aged 18–25 and other groups, those aged over 50 years old are less likely to suffer depression (OR = 0.448, 95%CI: 0.220–0.911, Cohen's d = −0.443). In comparison with young women, young men are more likely to develop depression. For example, compared with women aged 18–25, the prevalence of depression for men at the same age is higher (OR = 1.766, 95%CI: 1.219–2.560, Cohen's d = 0.314), peaking during their late 20s (OR = 2.024, 95%CI: 1.317–3.111, Cohen's d = 0.389) and then declining. For more details, Table 4 is demonstrated below. Sensitivity analysis was conducted by linear regression, and the results were consisted with the above (more detail can be seen in Figures 1, 2 and Table 5).

**Table 4:** Logistic regression analysis for the combined effect of gender and age on PTSS and depressive symptoms.

	**Model 4-PTSS**		**Model 5-Depression**	
**Variables**	**OR (95% CI)**	**Cohen's d**	**OR (95% CI)**	**Cohen's d**
Gender^*^age [Female (18–25)]				
Female (26–30)	1.505 (0.904, 2.505)	0.225	0.971 (0.630, 1.495)	−0.016
Female (31–40)	1.403 (0.835, 2.359)	0.187	1.004 (0.645, 1.563)	0.002
Female (41-50)	0.863 (0.450, 1.655)	−0.081	0.861 (0.506, 1.466)	−0.083
Female (≥51)	1.118 (0.521, 2.401)	0.061	0.448^*^ **(0.220, 0.911)**	−0.443
Male (18–25)	2.647^***^ **(1.711, 4.097)**	0.537	1.766^**^ **(1.219, 2.560)**	0.314
Male (26–30)	2.846^***^ **(1.725, 4.695)**	0.577	2.024^**^ **(1.317, 3.111)**	0.389
Male (31–40)	1.962^**^ **(1.181, 3.259)**	0.372	1.620^*^ **(1.050, 2.500)**	0.266
Male (41–50)	1.880^*^ **(1.050, 3.364)**	0.348	1.101 (0.658, 1.843)	0.053
Male (≥51)	1.323 (0.644, 2. 717)	0.154	0.777 (0.411, 1.467)	−0.139

The combine effect of gender and age was not significant in logistic regression analysis for insomnia, thus the results are not presented in this table; all confounding variables were controlled in the above models. The values of coefficients and 95% confidence interval in bold represent statistically significant at 0.05 level.

* p < 0.05,

** p < 0.01,

*** *p < 0.001*.

**Table 5:** Sensitivity analysis for the combined effect of gender and age on PTSS and depressive symptoms.

	**Model 6-PTSS**		**Model 7-Depression**	
**Variables**	**Coef. (Sta.Err)**	**Beta**	**Coef. (Sta.Err)**	**Beta**
Gender^*^age [Female (18–25)]				
Female (26–30)	0.521 (1.384)	0.010	−0.492 (0.920)	−0.014
Female (31–40)	−0.372 (1.456)	−0.008	−0.564 (0.968)	−0.017
Female (41–50)	−2.026 (1.711)	−0.029	−1.622 (1.138)	−0.035
Female (≥51)	−3.401 (2.025)	−0.037	–3.151 (1.346)^*^	−0.052
Male (18–25)	4.502 (1.251)^***^	0.079	1.766 (0.832)^*^	0.047
Male (26–30)	5.017 (1.458)^***^	0.083	3.545 (0.969)^***^	0.089
Male (31–40)	2.266 (1.460)	0.045	2.066 (0.971)^*^	0.063
Male (41–50)	0.7225 (1.715)	0.010	0.379 (1.140)	0.008
Male (≥51)	−1.976 (1.985)	−0.022	−2.313 (1.320)	−0.040

The combined effect of gender and age was not found significant in logistic regression analysis for insomnia, thus the results are not presented in this table; All confounding variables were controlled in the above models. The values of coefficients and 95% confidence interval in bold represent statistically significant at 0.05 level.

* p < 0.05,

*** *p < 0.001*.

## Discussion

This study attempted to reveal the mental health conditions among the population during the initial stage of the COVID-19 pandemic, and further to identify the combined effect of gender and age on the COVID-19 related mental health effects. Most importantly, this study found that the prevalence of PTSS, depression, and insomnia were 19.5, 26.9, and 19.6% respectively. Although no significant combined effect of gender and age was found in insomnia, PTSS, and depression closely related to gender-age interaction. Men in the late 20s were with relatively high PTSD symptoms, while the lowest prevalence of depression was found in women in the early 50s. At the same time, men aged 26–30 were more likely to get PTSS and depression. Besides, other factors related to PTSS, depression, and insomnia, in common or in particular, were confirmed either. Our findings identified factors associated with higher mental health symptoms so that they could be used to formulate psychological interventions to improve the mental health of vulnerable populations during the COVID-19 pandemic.

This study suggests that the public should pay greater attention to mental health conditions, as about one-fifth of the population (or over) has shown psychological symptoms. In the absence of traumatic events, the all-age prevalence for PTSS, depression, and insomnia in China are <1, 3.99, and 15% respectively (34–36). With the presence of disaster, the sweeping extent of the mental disorders also varies across traumatic types. An early review concludes the prevalence of PTSD at 5–10% among the general population after disasters (37), and later studies underline it as 8% in the Wenchuan earthquake (38), 8.6% after the flood (39), and <4% after terrorist attacks (40). The uncertain possibility of being infected leads to more PTSD symptoms among the general population, as 27% of individuals in Ebola-affected countries meet levels of clinical concerns for PTSD (41). Due to its huge disease burden in the general population, depression is the most prevalent mental disorder during the COVID-19 pandemic, and the number of people getting depression increases faster than after Hurricane Ike (42) and the 9–11 attack (43). It has to be noted that we estimate a slightly higher prevalence of PTSS and depression than prior studies, which were conducted about 10 days ahead (27, 28). Apart from the variance in sample distribution, the possible reason goes to the accumulative exposure under this pandemic. Communities continued to lockdown and almost all citizens were required to keep social distancing, especially people who could not return to their workplaces at the end of the New Year Holiday. Taking all the above comparisons, it is reasonable for this study to suggest that more attention is needed for mental health conditions under the COVID-19 pandemic.

Moreover, this study indicates an interesting reversal in the gender distribution of mental disorders. As noted by most trauma studies, women have higher incidence rates of mental health problems like PTSS and depression than their male counterparts (44, 45), explained partially by physiological differences or distinguished psychological mechanism (46). On the contrary, the evidence from this study supports that males are more possibly diagnosed with psychological disorders under the pandemic situation. An analogous conclusion could be seen in recent literature on COVID-19 (22) since the traditional gender roles and division is still prevalent in China (47). Chinese men as families' pillars have to take more psychological pressures for ensuring adequate supplies and the safety of the family during the COVID-19 pandemic, such as taking on family affairs with high exposure risk. In the meantime, the lack of strategies for men to cope with stress exacerbates their mental health disorders in COVID-19 scenarios. Previous studies find that men incline to reduce their pressure by resolving problems caused by stressors, while women turn to psychological adaptation (11, 48). However, with a universal lockdown policy, men who worry about their income could hardly find a way to solve the problem and thus experiencing high financial and living stress. Based on the prevalence of traditional gender role attitudes in China and the males' special strategy coping with stress, it is reliable for this study to claim that men express more mental health symptoms than women during the COVID-19 outbreak in China, therefore, releasing pressures on income and living is important to improve mental health.

Furthermore, a combined effect of gender and age is found upon PTSS and depression, indicating a different life-course expectation between men and women. Accordingly, previous studies show that women aged 26–30 may have the greatest depression and PTSD symptoms for the role burden and role conflict (49). For example, the responsibilities for taking care of families and troubles to balance work and family serve as a major source of psychological stress for young women. Greater psychological symptoms are assumed for women aged over 50, and the reasons are that changes in their reproductive ability, hormonal levels, and sympathetic responses tend to be risky (50). However, this study finds that them having the lowest level of depression. Perhaps, elderly women have stronger social support, lighter economic worries, and are under minimal media exposure. Comparatively, men suffer more from PTSD and depression in their early life in consideration of the family role and economic responsibility (51). Their mental health should be recognized as a social issue, with special attention paid to social problems such as unemployment, the familial disruption. Because of the similarities in the age distribution of psychological symptoms, we confirm that the income disruption raises the greatest negativity for both males and females, and figure out the age groups which should be concerned with priority. And the results also indicated that the gender difference in PTSS and depression could be amplified in young adulthood during the COVID-19, which partly supported our hypothesis. According to life course theory, younger adults usually enter into more new roles and statuses such as beginning marriage and becoming parents than elders, most of them have relative higher job strain and financial stress than older people who would exit from these roles and status (24). Therefore, young adults with these role transitions naturally suffer more financial pressure induced by the COVID-19 pandemic and lockdown, compare to older people. By combining the above explanation about gender difference that Chinese males as breadwinners usually had to bear most of these economic pressures, it could explain that the gender variation in PTSS and depression was magnified in the young adults. Therefore, policymakers should pay attention to these young males who suffer greater pressure because of their social roles and financial burden during this crisis.

Also, this study identifies the shared factors and the specific factors linked to PTSS, depression, and insomnia. Consistent with prior studies (52, 53), people with lower socioeconomic status and poorer health conditions, under more traumatic exposure, are found with greater vulnerabilities to PTSS, depression, and insomnia. Social support can help individuals mitigate PTSS and depression (54, 55). However, living with spouses may lead to greater mental health symptoms and it could be attributed to two aspects. On the one hand, married people are concerned not only for their own health but also for the health of their spouse in a pandemic, indicating an approximately 2-fold higher prevalence in mental disorders (56). Also, negative emotions may spread across individuals in a context full of unknown fears (57). On the other hand, married individuals have more concerns about the health of their families than their single counterparts (47). Besides, the significant variance in insomnia is not found in different age groups, while it is found in PTSS and depression. Possibly, greater hyper-arousal and sleep reactivity of young adults during the trauma counteracts the natural increasing prevalence of insomnia with age (58). The findings of this study implicate that interventions to improve mental health conditions of the population could be adapted with the types of psychopathologies and different sub-groups. It should be noted that health-related behaviors are also demonstrated to correlate with mental health conditions in the period of COVID-19 confinement, specifically, mental health symptoms could be mitigated by physical activity (59) or exacerbated through longer screen time (60). Also a study found that physical activity decreased while screen exposure time increased during the COVID-19 confinement (61). So we should consider reducing individuals' psychological symptoms by increasing their health-related behaviors in the mental health program during the lockdown and further control the variables related to health-related behaviors in future relevant studies.

## Limitations and Implications

It has to be noted that there are several limitations to this study. First, this study is based on a cross-sectional survey, indicating that only correlations rather than causal relationships between variables could be revealed. More longitudinal studies are needed to focus on causal relationships. Second, the representativeness of this sample to the general population may be biased. Since this study was conducted online and the elderly who did not have a smartphone might be excluded, the proportion of elderly respondents in this study is lower than it should be in the normal situation. With the adoption of snow-ball sampling, there may be a selection bias, leading to the underrepresentation of the general public and overrepresentation of individuals with specific status such as medical workers, students, and faculties. Overall, a community-based survey could be implemented in the future to avoid these limitations. Thirdly, PTSS, depression, and Insomnia are based on self-report scales. We used PCL-5 without a Criterion A component to assess PTSD symptoms. Clinical diagnosis should be used to increase the veracity of future research in this area.

Despite these limitations, this study is one of the few studies that focus on the interaction effect of age and gender on PTSS, depression, and insomnia among the Chinese general population during the early period of the COVID-19 outbreak. The findings of this study can help to examine the factors associated with the greatest mental health symptoms and provide implications for formulating psychological interventions. On one hand, mental health intervention programs, available psychological support resources, and the necessary economic grant should focus on groups with several special features, especially those who are likely to show two or more kind of mental health problems, such as people with post psychological problems, being male, suffering large impact on livelihood and with high exposure risks. On the other hand, young men take excessive stress because of their social roles and financial burden, which contribute more to mental health problems than exposure experiences. Thus, policy efforts must guarantee people's return to a safe and prejudice-free working environment and work efficiently with the necessary protective equipment.

## Conclusion

This study estimates that more or less one-fifth of the population have psychological symptoms during the COVID-19 outbreak. It has to be noted that males, especially young males suffer more from PTSS and depression. Additionally, people with lower socioeconomic status, poorer health conditions, and under extra traumatic exposure were found to be more susceptible to PTSS, depression, and insomnia. These findings are much supportive to screening the significant reasons linked with more mental health symptoms in current and future pandemic.

## Data Availability Statement

The raw data supporting the conclusions of this article will be made available by the authors, without undue reservation.

## Ethics Statement

The studies involving human participants were reviewed and approved by Peking University Medical Center. The patients/participants provided their written informed consent to participate in this study.

## Author Contributions

JG designed the study and conceived the manuscript. CL, DL, MF, JG, and YZ drafted the manuscript. XW, JFA, MS, and YW were involved in revising the manuscript. All authors were involved in writing the manuscript and approve of its final version.

## Conflict of Interest

The authors declare that the research was conducted in the absence of any commercial or financial relationships that could be construed as a potential conflict of interest.
